# Human genetic evidence challenges *NCOA1* as a monogenic obesity gene

**DOI:** 10.1016/j.xcrm.2026.103106

**Published:** 2026-10-08

**Authors:** Jérôme Delplanque, Audrey Leloire, Lauriane Le Collen, Hélène Loiselle, Bénédicte Toussaint, Emmanuel Vaillant, Frédéric Allegaert, Timothée Beke, Guillaume Charpentier, Sylvia Franc, Beverley Balkau, Michel Marre, Emma Henriques, Emmanuel Buse Falay, Mehdi Derhourhi, Philippe Froguel, Amélie Bonnefond

**Affiliations:** 1 Inserm/CNRS UMR 1283/8199, Institut Pasteur de Lille, EGID, Lille University Hospital, Lille, France; 2 University of Lille, Lille, France; 3 Biochemistry Department, Federative Institute of Biology, CHU, Toulouse, France; 4 Institute of Metabolic and Cardiovascular Diseases (I2MC), INSERM UMR 1297, Université Toulouse, Toulouse, France; 5 CERITD (Centre d’Étude et de Recherche pour l’Intensification du Traitement du Diabète), Evry, France; 6 Paris-Saclay University, Paris-Sud University, University of Versailles Saint-Quentin-en-Yvelines, Center for Research in Epidemiology and Population Health, Inserm U1018 Clinical Epidemiology, Villejuif, France; 7 Necker-Enfants Malades Institute, Inserm, University of Paris, Paris, France; 8 Ambroise Paré Clinic, Neuilly-sur-Seine, France; 9 Department of Metabolism, Imperial College London, Hammersmith Hospital, London, UK

**Keywords:** monogenic obesity, NCOA1, SRC1, precision medicine, functional genetics, melanocortin pathway

## Abstract

Obesity is a heterogeneous disease with a substantial genetic component. Rare pathogenic variants in genes of the leptin-melanocortin pathway can cause monogenic obesity and support targeted therapies such as setmelanotide, a melanocortin 4 receptor agonist. Pathogenic variants in *NCOA1*, encoding the nuclear receptor co-activator SRC-1, have recently been proposed as a cause of monogenic obesity through impaired leptin-induced POMC activation, leading to the inclusion of *NCOA1* variant carriers in the EMANATE setmelanotide trial. However, despite biological rationale, the contribution of *NCOA1* variants to human obesity remains elusive. Here, we assess *NCOA1* variants with case-control analyses in adults and children from the RaDiO study, combined with *in vitro* assays and replication in the UK Biobank. We find no association between pathogenic *NCOA1* variants and obesity or body mass index.

## Highlights

• *NCOA1* was proposed as an actionable form of monogenic obesity
• Here, functional genetics show no link between *NCOA1* variants and adiposity
• Results are replicated in ∼200,000 UK Biobank exomes
• Findings question the inclusion of *NCOA1* variant carriers in setmelanotide trials

## Graphical abstract

**Figure:**
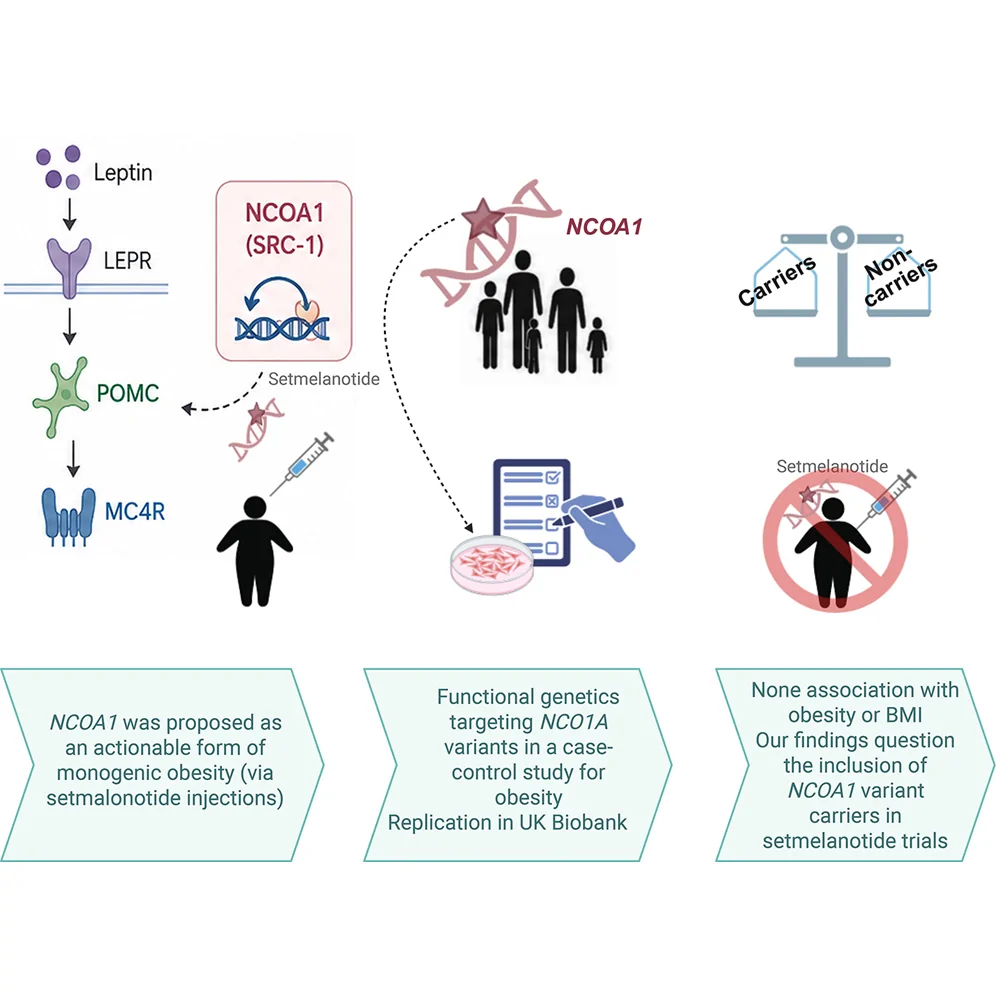

## Teaser

Delplanque et al. combine large-scale human genetics with functional analyses to reassess the role of *NCOA1* in obesity. Despite its biological plausibility within the melanocortin pathway, pathogenic *NCOA1* variants were not associated with adiposity, questioning their relevance for precision obesity therapies targeting this pathway.

## Introduction

Obesity has become one of the most pressing public-health challenges worldwide. In 2022, an estimated 2.5 billion adults (around 43% of the global adult population) presented with overweight, including about 890 million adults living with obesity, and the prevalence has more than doubled since 1990.^1^ Obesity is usually defined clinically with the body mass index (BMI): in adults, obesity corresponds to a BMI ≥30 kg/m^2^ (and to age- and sex-adjusted BMI-for-age cut-offs in children and adolescents).

Obesity has a strong genetic component. In the general population, the predominant form is polygenic obesity, in which hundreds of common variants, each contributing only modestly to BMI and body fat accumulation, act additively and in combination with environmental and lifestyle exposures.^2^^,^^3^ In contrast, monogenic obesity refers to rare, highly penetrant variants in single genes that have a large effect on energy balance, typically causing severe, early-onset obesity, often before 5 years of age, frequently with hyperphagia and sometimes with additional endocrine or developmental features.^4^ Many of these monogenic forms result from pathogenic variants in the components of the leptin-melanocortin pathway (e.g., *LEP*, *LEPR*, *POMC*, *PCSK1*, *MC4R*, *SIM1*, and *MRAP2*), which plays a central role in hypothalamic control of appetite.^4^ Importantly, several monogenic obesity syndromes are now considered clinically actionable. Actionability means that identifying the causal genetic defect can directly inform a specific, evidence-based intervention that meaningfully improves outcomes (e.g., weight, metabolic health, or associated complications). Classic examples include recombinant leptin therapy for congenital leptin deficiency (*LEP*)^5^ and more recently, targeted use of the melanocortin-4 receptor (MC4R) agonist setmelanotide in individuals with bi-allelic pathogenic variants in *POMC*, *PCSK1*, or *LEPR*.^6^

*NCOA1* encoding nuclear receptor co-activator 1 (also known as steroid receptor coactivator-1, SRC-1), has been recently identified as a candidate gene for monogenic obesity.^7^ In a unique, large sequencing study of individuals with severe early-onset obesity, rare heterozygous *NCOA1* variants were enriched among cases and were shown functionally to impair co-activator activity, with carriers often presenting with hyperphagia and reduced satiety.^7^ Experimental mouse models supported a key role for NCOA1 in hypothalamic regulation of energy balance: *Ncoa1* was expressed in pro-opiomelanocortin (POMC) neurons of the arcuate nucleus, and selective deletion of *Ncoa1* in these neurons reduced *Pomc* expression, leading to hyperphagia, increased fat mass, and susceptibility to diet-induced obesity in mice.^7^ These data positioned NCOA1 as a key modulator of the leptin-melanocortin axis within the hypothalamus. On this basis, patients with monoallelic pathogenic *NCOA1* variants have been included in ongoing clinical trials of setmelanotide (i.e., the phase 3 EMANATE study), which evaluates the efficacy and safety of this MC4R agonist in individuals with obesity and pathogenic variants in the MC4R pathway. However, although the biological rationale is strong, the relationship between *NCOA1* variants and obesity pathogenesis in humans has not yet been fully clarified.

Here, we report a case-control study of obesity focused on *NCOA1* that was conducted in both adults and children from the RaDiO study, including functional investigation of *NCOA1* variants, with independent validation in the UK Biobank. No association was detected between pathogenic or likely pathogenic *NCOA1* variants (including those deleterious based on our *in vitro* assays) and either obesity or BMI.

## Results

### Identification and functional characterization of rare *NCOA1* variants in 6,491 participants

Coding exons of *NCOA1* (GenBank: NM_003743.5) were sequenced in case-control studies of adult and childhood obesity, comprising a total of 6,491 participants. We identified 80 rare variants, including 1 canonical splice-site variant (c.2887-2del), 2 in-frame deletions (p.Ser999del and p.Pro1157_Pro1160del), and 77 missense variants (Table S1). Of the 80 variants identified, 19 were novel, with no entries in the Genome Aggregation Database (gnomAD: v.4.1.0; Table S1). To assess their functional impact, each missense variant or deletion was tested in Neuro-2A cells by transfecting the wild-type or mutant *NCOA1* construct together with *STAT3*, *LEPR*, and *POMC* promoter reporter plasmids. By luciferase assays, we then quantified the ability of each *NCOA1* variant to activate *POMC* expression in response to leptin stimulation. Following leptin stimulation, we identified 14 missense *NCOA1* variants that significantly reduced *POMC* expression (Figure 1), including three novel variants: p.Thr188Ile, p.Val390Phe, and p.Leu1415Val (Table S1), through unpaired Mann-Whitney tests, with the “WT Leptin” condition used as the reference control. The other tested variants did not have any effect on *POMC* expression upon leptin stimulation (Figure S1).

**Figure 1:**
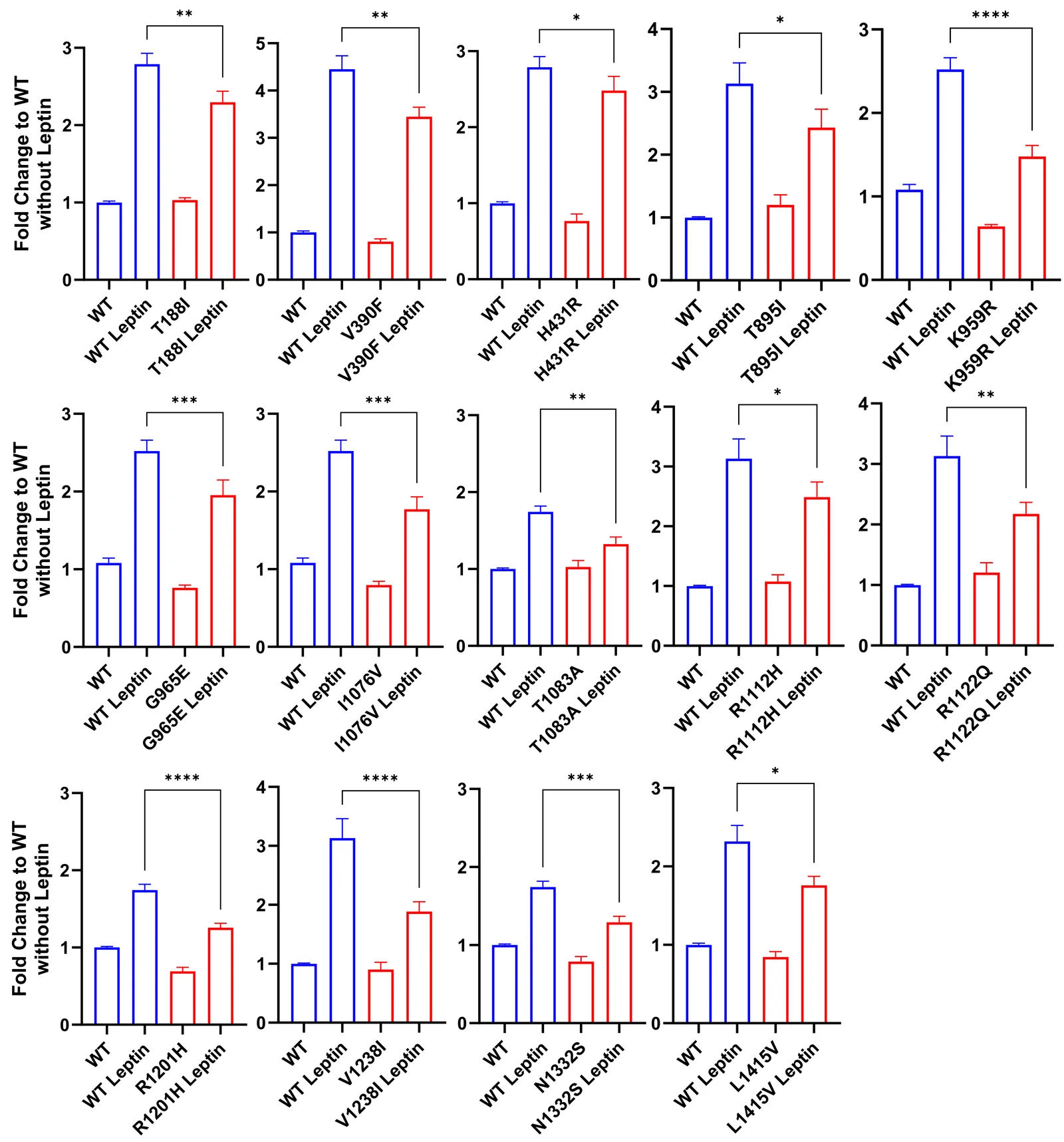
Effect of loss-of-function *NCOA1* variants on *POMC* expression in response to leptin in Neuro-2A cells Neuro-2A cells were transiently transfected with a plasmid encoding β-galactosidase for normalization, together with the *NCOA1* expression plasmid (wild-type [WT] or mutant) and the *STAT3*, *LEPR*, and *POMC* promoter reporter constructs. Data are fold changes to luciferase activity of WT condition without leptin (mean ± SEM). All experiments were performed in technical triplicate and each experiment was repeated at least seven times (i.e., with a minimum of seven biological replicates). *p* values were calculated related to “WT Leptin” by unpaired Mann-Whitney tests. ∗*p* value <0.05, ∗∗*p* value <0.01, ∗∗∗*p* value <0.001, ∗∗∗∗*p* value <0.0001.

### Association between pathogenic *NCOA1* variants and adiposity

We next assessed the pathogenicity of each variant with the American College of Medical Genetics and Genomics (ACMG) guidelines, incorporating the strong PS3 criterion, which corresponds to functional evidence consistent with a deleterious effect.^8^ We found 12 monoallelic pathogenic or likely pathogenic (P/LP) variants (Table S1). The majority of the missense P/LP variants were located in key functional domains of NCOA1 (Figure 2). Variants p.Thr188Ile and p.Leu1415Val were located within intrinsically disordered regions, while p.Val390Phe was found in a disordered segment suspected to interact with STAT3 according to UniProt (UniProt: #Q15788; Figure 2). Variants p.Thr895Ile, p.Gly965Glu, and p.Lys959Arg were located in a region predicted to interact with CREBBP; notably, p.Lys959Arg also fell within the activation domain 1 (AD1) of NCOA1 and was predicted to undergo ubiquitination according to UniProt (Uniprot: #Q15788). In addition, p.Arg1201His was positioned within activation domain 2 (AD2) of NCOA1 (Figure 2).^9^^,^^10^

**Figure 2:**
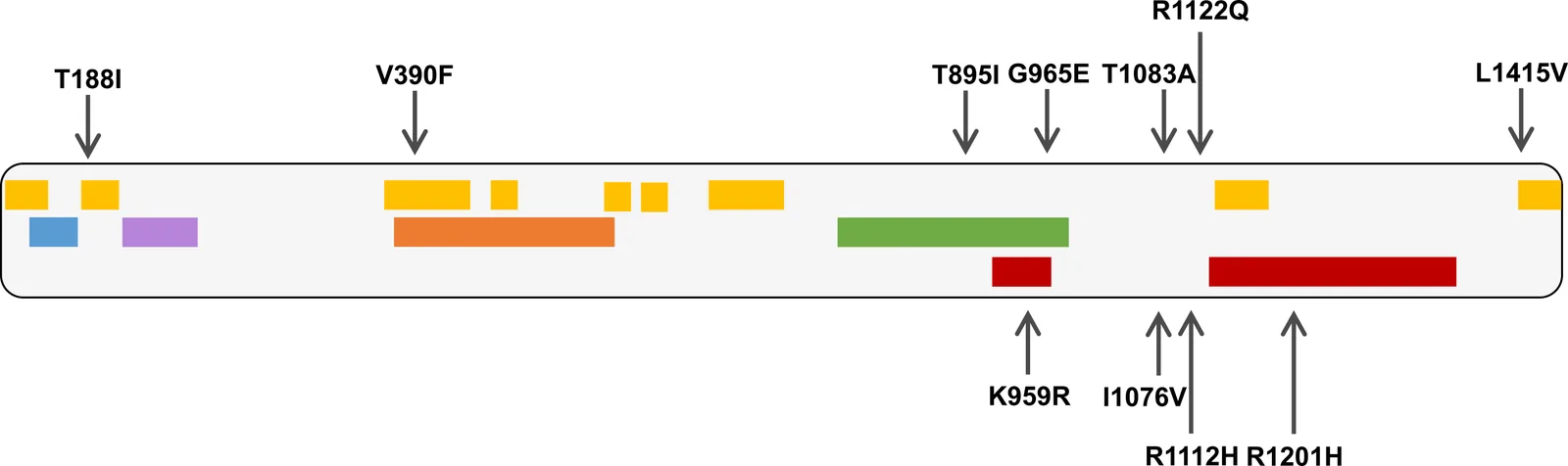
Location of P/LP missense variants in *NCOA1* identified in the RaDiO study Yellow domains represent disordered regions; the blue domain represents the bHLH region; the purple domain represents the PAS region; the orange domain represents the interaction with STAT3; the green domain represents the interaction with CREBBP; according to UniProt (#Q15788). The red regions represent activation domain 1 (AD1, *left*) and activation domain 2 (AD2, *right*) of NCOA1, according to Sheppard et al.^9^ and Segovia and Tepes.^10^

We next assessed the presence of obesity among carriers of monoallelic P/LP *NCOA1* variants. In both adults and children, exactly half of the carriers presented with obesity and the other half had normal weight (8/16 overall). To analyze the statistical effect of P/LP *NCOA1* variants on obesity risk, variants were analyzed jointly as a single cluster with the MiST method,^11^ as previously used by us.^12^ MiST analyses were adjusted for age, sex, and ancestry. We found that the burden of P/LP *NCOA1* variants was not significantly associated with obesity risk (Table 1). Notably, the p.Lys959Arg P/LP variant, i.e., the one showing the strongest inhibitory effect on *POMC* expression among P/LP variants (Figure 1) and located within key functional domains of NCOA1 (Figure 2), was identified in controls only, i.e., one adult with normal weight and one child with normal weight.

**Table 1:** Association between P/LP *NCOA1* variants and obesity traits in RaDiO and UK Biobank

Study	Trait	*n*	% (ratio) or median [IQR] of the trait in carriers	% (ratio) or median [IQR] of the trait in non-carriers	OR [CI]	Π ± SE	*p*
RaDiO adults	obesity	4,405	50% (3/6)	35% (1,520/4,399)	1.6 [0.56–5.1]	–	0.36
RaDiO children	obesity	2,086	50% (5/10)	50% (1,039/2,076)	1.2 [0.45–3.3]	–	0.75
UK Biobank	BMI (kg/m^2^)	187,628	27 [23–29]	27 [24–30]	–	0.091 ± 0.054	0.091
UK Biobank	obesity	107,153	50% (4/8)	42% (45,480/107,209)	1.6 [0.35–7.1]	–	0.54

Association analyses were performed with the MiST method adjusted for age, sex, and ancestry. BMI was log-transformed before analysis. BMI, body mass index; CI, confidence interval; IQR, interquartile range; π, effect of the burden; OR, odds ratio; SE, standard error.

### Replication study with the UK Biobank

Subsequently, we extracted null P/LP *NCOA1* variants from 200K exome-sequenced participants in the UK Biobank (Table S2) and assessed the effect of their burden on BMI and obesity risk. The median BMI was identical between carriers and non-carriers, and, consistent with our case-control studies, 50% of carriers had obesity while the remaining 50% had normal weight (Table 1). With MiST analyses adjusted for age, sex, and ancestry, the burden of P/LP *NCOA1* variants was not significantly associated with obesity risk or BMI (Table 1).

## Discussion

We did not identify any evidence supporting an impact of heterozygous P/LP *NCOA1* variants on obesity. This result contradicts the only previous report that suggested that this gene caused a monogenic form of obesity.^7^ To reach this conclusion, we combined two complementary approaches: (1) functional genetics in the RaDiO study allowing us to detect P/LP *NCOA1* missense variants with deleterious *in vitro* effect and (2) population-scale genetic analyses, assessing the effect of *NCOA1* null variants in the UK Biobank. Both lines of evidence converge toward the absence of a measurable impact of heterozygous *NCOA1* loss-of-function variants on increased BMI or obesity risk.

Notably, p.Leu1376Pro was the only variant shared between the initial publication by *Yang* et al.^7^ and our RaDiO study. This variant has a relatively high minor allele frequency (∼10^–4^) in gnomAD with 154 carriers reported (Table S1). While *Yang* et al. reported this variant as deleterious, our assays did not support this conclusion (Figure S1). Although we cannot completely exclude a difference in sensitivities between *in vitro* assays, it is important to highlight that this variant does not lie within any functionally characterized domain of NCOA1 (Figure 2), which already raises questions about its possible functional relevance. Furthermore, in the RaDiO study, 2 out of 4 carriers of this variant had a normal body weight, and 13 out of 14 carriers in the UK Biobank did not have obesity, reinforcing its low penetrance and the absence of a consistent obesity phenotype among carriers.

Together, these findings make it very unlikely that heterozygous P/LP *NCOA1* variants can cause monogenic obesity. Therefore, it is difficult to justify the continued inclusion of NCOA1 in clinical trials for setmelanotide, as the current evidence for causality remains limited and inconsistent. We previously showed that heterozygous P/LP variants in both *POMC* and *LEPR* genes did not cause monogenic obesity (despite being included in the phase 3 EMANATE trial),^13^^,^^14^ and that only complete loss-of-function heterozygous variants in *PCSK1* caused monogenic obesity potentially responsive to setmelanotide.^15^ Together, these data strongly call for caution before extending the use of expensive drugs with notable side effects beyond their established indication, i.e., the treatment of well-documented cases of severe early-onset obesity due to defects in the leptin-melanocortin pathway beyond MC4R.

### Limitations of the study

We acknowledge that our study has several limitations. First, our *in vitro* investigations were restricted to luciferase assays assessing the effects of the variants on the *POMC* promoter in response to leptin stimulation. Although we deliberately focused on the same pathway investigated in the original study,^7^ we cannot exclude the possibility that these variants may affect other biological mechanisms or signaling pathways.

In addition, we cannot formally rule out insufficient sensitivity of our experimental approach. However, it is noteworthy that null variants themselves were not associated with adiposity, further supporting our findings.

Finally, we did not investigate the effects of the variants on food intake, satiety, energy expenditure, body composition, or related metabolic phenotypes. Instead, we deliberately focused on BMI and obesity risk, in line with the ongoing clinical trial, for which the primary outcome is weight loss.

## Resource availability

### Lead contact

Further information and requests for resources and reagents should be directed to and will be fulfilled by the lead contact, Amélie Bonnefond (amelie.bonnefond@inserm.fr).

### Materials availability

This study did not generate new unique reagents.

### Data and code availability

All data reported in this paper will be shared by the lead contact upon request. Any additional information required to reanalyze the data reported in this paper is available from the lead contact upon request. This paper does not report original code. All analyses were performed using R (RStudio) with publicly available, well-established R packages and did not involve novel algorithms or non-standard analytical workflows.

## Acknowledgments

We are grateful to all individuals included in the RaDiO study and UK Biobank. The authors would like to thank Anne-Sophie Ledoux and Vincent Massy for technical assistance. This study was supported by grants from the 10.13039/501100001665French National Research Agency (ANR-10-LABX-46 [10.13039/100017228European Genomic Institute for Diabetes] and ANR-10-EQPX-07-01 [LIGAN-PM]), the European Union’s Horizon Europe Research and Innovation Programme (OBELISK grant agreement no. 101080465), and the National Center for Precision Diabetic Medicine – PreciDIAB, which is jointly supported by the 10.13039/501100001665French National Agency for Research (ANR-18-IBHU-0001), the 10.13039/501100000780European Union (FEDER) 20001891/NP0025517, the 10.13039/501100014161Hauts-de-France Regional Council, and the European Metropolis of Lille (MEL 2019_ESR_11). A.B. is supported by the 10.13039/501100000781European Research Council (OπO 101043671). We thank the France Génomique consortium (ANR-10-INBS-009).

## Author contributions

Conceptualization, J.D., A.L., P.F., and A.B.; methodology, J.D., A.L., L.L.C., H.L., B.T., E.V., F.A., T.B., E.H., E.B.F., M.D., and A.B.; software, E.H., E.B.F., and M.D.; formal analysis, J.D., A.L., E.H., E.B.F., M.D., and A.B.; data collection, G.C., S.F., B.B., M.M., P.F., and A.B.; data curation, J.D., A.L., E.H., E.B.F., M.D., and A.B.; writing – original draft, A.B.; writing – review and editing, J.D., A.L., L.L.C., H.L., B.T., E.V., F.A., T.B., G.C., S.F., B.B., M.M., E.H., E.B.F., M.D., P.F., and A.B.; visualization, J.D., A.L., and A.B.; funding acquisition, P.F. and A.B.

## Declaration of interests

The authors declare no competing interests.

## STAR★Methods

### Key resources table

**Table:** 

REAGENT or RESOURCE	SOURCE	IDENTIFIER
**Chemicals, peptides,and recombinant proteins**		

Dulbecco’s modified Eagle’s medium	Pan Biotech	Cat# P04-00509
Fetal Bovine Serum	Gibco	Cat# A5256701
Penicillin - Streptomycin	Gibco	Cat# 15140122
Leptin	PreproTech	Cat# 300–27

**Critical commercial assays**		

Magnetofection – NeuroMag Transfection Reagent	OZ Biosciences	Cat# NM51000
Luciferase Assay System	Promega	Cat# E1510
Quickchange II XL site-directed mutagenesis kit	Agilent Technologies	Cat# 200522
NimbleGen SeqCap EZ Choice XL target enrichment	Roche	Cat# 6266398001
HiSeq 4000 PE Cluster Kit	Illumina	Cat# PE-410–1001
HiSeq 4000 SBS Kit (300 cycles)	Illumina	Cat# FC-410–1003

**Deposited data**		

Genome Aggregation Database (gnomAD; v4.1.0)	Gudmundsson el al.^16^	https://gnomad.broadinstitute.org/
Human reference genome NCBI build 37, GRCh37	Genome Reference Consortium	https://www.ncbi.nlm.nih.gov/projects/genome/assembly/grc/human/

**Experimental models: Cell lines**		

Mouse: Neuro-2A cells	Merck Group	Cat# 89121404-1VL

**Recombinant DNA**		

Plasmid: *NCOA1* (NM_003743.5)	Sartorius Polyplus	Tailor-designed plasmid
Plasmid: *NCOA1*-p.Ser999del (NM_003743.5)	Sartorius Polyplus	Tailor-designed plasmid
Plasmid: *NCOA1*-p.Pro1157_Pro1160del (NM_003743.5)	Sartorius Polyplus	Tailor-designed plasmid
Plasmid: *LEPR* (NM_002303.6)	Twist Bioscience	Tailor-designed plasmid
Plasmid: *STAT3* (NM_139276.3)	Origene	Cat# RC215836
Plasmid: *POMC* promoter: ∗ (−949 to +416 relative to the transcription start site) ∗ pGl3 Basic Vector	Twist Bioscience Promega	Tailor-designed DNA fragment Cat# E1751
pSV-β-Galactosidase Control Vector	Promega	Cat# E1081

**Software and algorithms**		

ACMG	Richards et al.^8^	N/A
MiST	Sun et al.^11^	N/A
REVEL	Ioannidis et al.^17^	https://sites.google.com/site/revelgenomics/
Ensembl Variant Effect Predictor (VEP) tool version 103	McLaren et al.^18^	https://www.ensembl.org/info/docs/tools/vep/script/vep_options.html
bcl2fastq Conversion Software (v2.17)	Illumina	N/A
Burrows-Wheeler Aligner (v0.7.13)	Li et al.^19^	N/A
Genome Analysis ToolKit (GATK; v3.3)	McKenna et al.^20^	N/A

### Experimental model and study participant details

#### Study participants

In RaDiO (***Ra****re variants involved in* ***Di****abetes and* ***O****besity*), we analyzed two case-control studies: one focused on adult obesity (1,523 cases with obesity versus 2,882 controls with normal weight) and another dedicated to childhood obesity (1,044 cases with childhood obesity versus 1,042 controls with normal weight). Participants were from several population studies.^15^ All population studies followed ethical principles defined in the Helsinki declaration (revised in 1996), and they were approved by local ethical committees. All participants signed an informed consent form. Oral assent from children/adolescents was obtained and parents (or legal guardians) signed and informed consent form. Study enrollment was voluntary.

In UK Biobank, we analyzed up to 187,628 participants, with available exome sequencing data. UK Biobank has approval from the North West Multi-centre Research Ethics Committee as a Research Tissue Bank approval. UK Biobank individual-level, phenotypic data and whole exome sequencing data used in the present work were obtained under application #67575.

In participants older than 18 years, normal weight was defined as BMI <25 kg/m^2^, and obesity as BMI ≥30 kg/m^2^. In children and adolescents younger than 18 years, normal weight was defined as BMI-for-age <85^th^ percentile and obesity as BMI-for-age ≥95^th^ percentile.^21^

### Method details

#### *NCOA1* sequencing and variant detection

In the RaDiO study, DNA sequencing of *NCOA1* (NM_003743.5) and detection of variants were performed by next-generation sequencing as previously described.^12^ Briefly, NimbleGen SeqCap EZ Choice XL target enrichment (Roche, Pleasanton, USA) was performed according to the manufacturer’s protocol for next-generation sequencing on the HiSeq 4000 system (Illumina, San Diego, USA), using a paired-end 2 × 150 bp protocol. The demultiplexing of sequence data was performed using bcl2fastq Conversion Software (Illumina; v2.17). Sequence reads were then mapped to the human genome (hg19/GRCh37) using Burrows-Wheeler Aligner (v0.7.13). The variant calling was performed using Genome Analysis ToolKit (GATK; v3.3). Only variants with a coverage higher than 8 reads were kept for further analyses. The annotation of variants was performed using the Ensembl Variant Effect Predictor (VEP) tool version 103. The study was focused exclusively on rare coding variants (i.e., with a minor allele frequency [MAF] < 5% in any ancestry according to GnomAD [v4.1.0]). All these rare variants had a QUAL score higher than 50. Furthermore, no variant had more than 5% missing genotype (with a coverage below 8 reads or a QUAL score below 50) across the participants.

In UK Biobank, we used exome data from pVCF format (field #23156). Only variants with a coverage higher than 10 reads and quality GQ score higher than 20 were kept for further analyses. Annotation of variants in *NCOA1* (NM_003743.5) was done using the Ensembl VEP tool version 103. Subsequently, the analysis was focused on pathogenic or likely pathogenic (P/LP) variants only (see below for further details). No P/LP variant had more than 1% missing genotype (i.e., with a coverage below 10 reads or a GQ score below 20) across the participants.

#### Variant pathogenicity

To assess the pathogenicity of the variants in *NCOA1*, we used the standards and guidelines of the American College of Medical Genetics and Genomics (ACMG).^8^ In particular, variants addressing the very strong pathogenic criterion PVS1 were null (e.g., canonical splice-site, frameshift, stop-gain variants); variants addressing the strong pathogenic criterion PS3 were deleterious according to our *in vitro* assays; and variants addressing the moderate pathogenic criterion PM2 had a minor allele frequency below 0.00001 in GnomAD (v4.1.0). None variants addressed the supporting pathogenic criterion PP3 according to the REVEL score (threshold at 0.75).^17^

#### Plasmids

The wild-type *NCOA1* vector (NM_003743.5) was produced by Sartorius Polyplus using a modular DNA fragment assembly strategy and cloned into a pEF1 expression vector. The two vectors with *NCOA1* deletions (c.2995_2997del/p.Ser999del and c.3468_3479del/p.Pro1157_Pro1160del) were also built by Sartorius Polyplus using the same technology. *NCOA1* construct including each missense variant were generated using the Quickchange II XL site-directed mutagenesis kit (Agilent Technologies). Each novel plasmid was checked by Sanger sequencing after mutagenesis. The *LEPR* vector (NM_002303.6) was produced by Twist Bioscience and delivered in a pEF1 expression vector. The *STAT3* plasmid (NM_139276.3) was purchased from Origene. A fragment of the human *POMC* promoter (−949 to +416 relative to the transcription start site) was synthesized by Twist Bioscience with BglII at the −949 end and HindIII at the +416 end, and cloned into the pGL3-Basic vector (Promega).

#### *In vitro* assays

Neuro-2A cells (Mouse neuroblastoma; Merck) were cultured in Dulbecco’s modified Eagle’s medium supplemented with 10% fetal bovine serum, 100 IU/mL penicillin and 100 ng/mL streptomycin. Cells were seeded at 250,000 cells per well in 24-well plates and incubated overnight. They were then transfected with 100 ng/well of the *POMC*-luciferase reporter plasmid, 200 ng/well of the *LEPR* vector, 200 ng/well of the *STAT3* vector, 75 ng/well of the β-galactosidase plasmid and 600 ng/well of either wild-type *NCOA1* or mutant *NCOA1* plasmid, using Magnetofection with the NeuroMag Transfection Reagent (OZ Biosciences). The next day, the medium was replaced with serum-free medium supplemented with 100 IU/mL penicillin and 100 ng/mL streptomycin for overnight serum starvation. 48 h after transfection, the Neuro-2A cells were treated with or without leptin (0.2 μg/mL; PeproTech Proteins) for 20 min and then kept cultured in fresh starvation medium for 6 h. The cells were then lysed using 200 μL of luciferase cell culture lysis buffer (Promega). Luminescence was quantified by adding 25 μL of the luciferase assay system reagent (Promega) to 40 μL of lysate. The signal was recorded using a GloMax Discover luminometer (Promega). β-galactosidase activity was measured after incubation of 40 μL of lysate for 5 min with 100 μL of homemade buffer [0.479 g NaHPO4, 1.060 g NAH2PO4:2H2O, 0.5 mL KCl 2 M, 0.1 mL 1 M MgCl2, q.s. to 100 mL of water; extemporaneous addition of 325 μL β-mercaptoethanol and 25 mL of 4 mM *ortho*-nitrophenyl-β-galactoside] and was read using the Glomax Discover luminometer (Promega). Luciferase activity was calculated as relative luminescence units (RLUs) normalized against β-galactosidase activity. All experiments were performed in technical triplicate and each experiment was repeated at least seven times (i.e., with a minimum of seven biological replicates).

### Quantification and statistical analyses

#### Statistical analyses for genetic association studies

In RaDiO and UK Biobank, the P/LP *NCOA1* variants were analyzed as single clusters using the MiST method,^11^ as previously described.^12^ MiST provides a score statistic S(π) for the mean effect (π) of the cluster, and a score statistic S(τ) for the heterogeneous effect (τ) of the cluster. The MiST tests were adjusted for age, sex and ancestry (i.e., genetic principal components PC1-PC5). In UK Biobank, BMI was log-transformed before analysis. As none of the association studies had significant heterogeneity, we only showed the *p*-values associated with the mean effect (π) of the cluster. These statistical analyses were performed using R software.

#### Statistical analyses for *in vitro* studies

*p*-values assesing the ability of each *NCOA1* variant to activate *POMC* expression following leptin stimulation (i.e., “Variant XXX Leptin”) were calculated relative to the leptin-stimulated control condition (i.e., “WT Leptin”) using unpaired Mann-Whitney tests.

## Supplemental information

Document S1. Figure S1 and Tables S1 and S2

Document S2. Article plus supplemental information
