# Supplementary material for: Human genetic evidence challenges NCOA1 as a monogenic obesity gene
Source: Cell Rep Med. 2026 Oct 8;7(10):103106. doi: 10.1016/j.xcrm.2026.103106 (PMC13710694; doi:10.1016/j.xcrm.2026.103106)
Supplement: Supplementary file 1 [file mmc1.pdf]

Cell Reports Medicine, Volume *7*

Supplemental information

Human genetic evidence challenges *NCOA1* as a monogenic obesity gene

Jérôme Delplanque, Audrey Leloire, Lauriane Le Collen, Hélène Loiselle, Bénédicte Toussaint, Emmanuel Vaillant, Frédéric Allegaert, Timothée Beke, Guillaume Charpentier, Sylvia Franc, Beverley Balkau, Michel Marre, Emma Henriques, Emmanuel Buse Falay, Mehdi Derhourhi, Philippe Froguel, and Amélie Bonnefond

Supplementary Table 1. ***NCOA1*** **variants detected in the RaDiO study**

Nucleotide change (NM_003743.5)	Amino acid change	MAC	Pathogenicity	MAF GnomAD (v4.1.0)	LOF
c.92C>T	p.Thr31Met	1		2.72197E-05	
c.269C>A	p.Thr90Asn	1		0	
c.563C>T	p.Thr188Ile	2	P/LP	0	×
c.619C>T	p.Pro207Ser	1		1.85875E-06	
c.628G>A	p.Glu210Lys	1		7.00107E-05	
c.763G>T	p.Ala255Ser	2		0	
c.811A>G	p.Lys271Glu	1		0	
c.883T>A	p.Cys295Ser	1		0	
c.959G>A	p.Arg320His	1		2.41742E-05	
c.1168G>T	p.Val390Phe	1	P/LP	0	×
c.1259G>A	p.Arg420His	4		9.47964E-05	
c.1292A>G	p.His431Arg	3		0.000395268	×
c.1316G>A	p.Gly439Glu	1		3.09757E-06	
c.1337G>T	p.Gly446Val	1		0	
c.1342C>A	p.Gln448Lys	1		8.67378E-06	
c.1402T>G	p.Ser468Ala	1		3.03582E-05	
c.1415A>G	p.Asn472Ser	2		5.26581E-05	
c.1436C>A	p.Thr479Lys	1		0	
c.1445C>G	p.Ser482Cys	2		5.4521E-05	
c.1459A>T	p.Met487Leu	1		0	
c.1490T>C	p.Leu497Ser	2		2.04449E-05	
c.1509G>A	p.Met503Ile	2		6.19556E-06	
c.1535T>G	p.Ile512Ser	1		6.19495E-07	
c.1606A>G	p.Ile536Val	1		6.1951E-07	
c.1622T>C	p.Leu541Ser	2		9.04542E-05	
c.1646A>G	p.Asn549Ser	1		0.000345683	
c.1678G>A	p.Val560Ile	1		1.23913E-05	
c.1700A>G	p.Gln567Arg	1		9.29301E-06	
c.1716A>T	p.Arg572Ser	64		0.001398808	
c.1724T>C	p.Ile575Thr	2		5.0799E-05	
c.1811A>G	p.Asp604Gly	1		7.43386E-06	
c.1949T>C	p.Ile650Thr	1		9.91185E-06	
c.1973T>C	p.Leu658Pro	2		0	
c.2011T>G	p.Ser671Ala	1		4.39876E-05	
c.2056C>T	p.Arg686Trp	1		1.85858E-06	
c.2191A>G	p.Ile731Val	1		1.85861E-06	
c.2245C>A	p.Leu749Ile	1		7.43478E-06	
c.2252A>G	p.Tyr751Cys	3		1.30094E-05	
c.2323G>C	p.Val775Leu	1		2.29232E-05	
c.2329A>G	p.Lys777Glu	1		3.09774E-06	
c.2339A>G	p.Gln780Arg	2		4.95635E-06	
c.2368C>G	p.Pro790Ala	1		0	
c.2384C>T	p.Thr795Ile	1		4.24825E-05	
c.2434G>A	p.Asp812Asn	1		3.22231E-05	
c.2489T>C	p.Leu830Ser	2		0	
c.2516C>T	p.Ala839Val	1		6.3191E-05	
c.2569G>A	p.Ala857Thr	26		0.000527905	
c.2659G>T	p.Asp887Tyr	1		1.3656E-05	
c.2684C>T	p.Thr895Ile	1	P/LP	2.48378E-06	×
c.2719C>G	p.Gln907Glu	1		6.24589E-07	
c.2725A>G	p.Ile909Val	1		0	
c.2753G>A	p.Cys918Tyr	4		9.91885E-06	
c.2876A>G	p.Lys959Arg	2	P/LP	2.54399E-05	×
c.2881G>T	p.Val961Phe	1		1.24211E-06	
c.2887-2del	-	2	P/LP	6.21531E-07	-

c.2887G>C	p.Gly963Arg	1		2.48493E-06	
c.2894G>A	p.Gly965Glu	2	P/LP	1.85977E-06	×
c.2995_2997del	p.Ser999del	1		2.41628E-05	
c.3020C>T	p.Pro1007Leu	1		0	
c.3143C>T	p.Pro1048Leu	2		1.73474E-05	
c.3209A>G	p.His1070Arg	1		6.19922E-07	
c.3226A>G	p.Ile1076Val	1	P/LP	5.14326E-05	×
c.3247A>G	p.Thr1083Ala	1	P/LP	2.47837E-06	×
c.3265A>G	p.Asn1089Asp	1		1.73497E-05	
c.3335G>A	p.Arg1112His	1	P/LP	1.23947E-05	×
c.3365G>A	p.Arg1122Gln	1	P/LP	1.73475E-05	×
c.3368A>G	p.Gln1123Arg	1		0	
c.3468_3479del	p.Pro1157_Pro1160del	1		0	
c.3602G>A	p.Arg1201His	1	P/LP	3.71703E-06	×
c.3662T>C	p.Ile1221Thr	1		0	
c.3712G>A	p.Val1238Ile	2		0.000125661	×
c.3725A>G	p.Glu1242Gly	2		0	
c.3814C>T	p.Pro1272Ser	564		0.021742955	
c.3914C>T	p.Thr1305Met	1		1.61097E-05	
c.3991A>G	p.Met1331Val	1		6.1951E-07	
c.3995A>G	p.Asn1332Ser	20		0.002281956	×
c.4066A>C	p.Ile1356Leu	6		8.49313E-05	
c.4127T>C	p.Leu1376Pro	6		9.54124E-05	
c.4243C>G	p.Leu1415Val	1	P/LP	0	×
c.4286C>G	p.Ala1429Gly	1		1.85975E-06	

Orange (in column "Amino acid change") highlights null variants (ACMG PVS1 criterion); yellow (in column "MAF GnomAD") highlights variants with a MAF below 0.0001 in GnomAD v4.1.0 (ACMG PM2 criterion); pink (in column LoF) highlights loss-of-function variants according to our *in vitro* assays (ACMG PS3 criterion).
LOF, loss-of-function variants according to luciferase assays; ***MAC***, minor allele count; ***MAF***, minor allele frequency; ***N***, neutral; ***P/LP***, pathogenic or likely pathogenic variant.

Supplementary Table 2. Null monoallelic *NCOA1* variants detected in the UK Biobank

Nucleotide change (NM_003743.5)	Amino acid change	MAC in UK Biobank	MAC in GnomAD (v4.1.0)
c.90-12_92del		1	1
c.713-1G>C		1	1
c.914_918del	p.Arg305ThrfsTer23	1	1
c.2142_2143dup	p.Ala715ValfsTer8	1	1
c.2886+6_2886+9del		5	-
c.2887-2_2887-1del		3	6
c.3085C>T	p.Arg1029Ter	1	1
c.3163C>T	p.Arg1055Ter	1	1
c.3304-1G>T		1	1
c.3766del	p.Val1256CysfsTer27	1	1
c.4065+1G>T		1	8

MAC, minor allele count

Supplementary Figure 1. Effect of neutral *NCOA1* variants on *POMC* expression in response to leptin in Neuro-2A cells

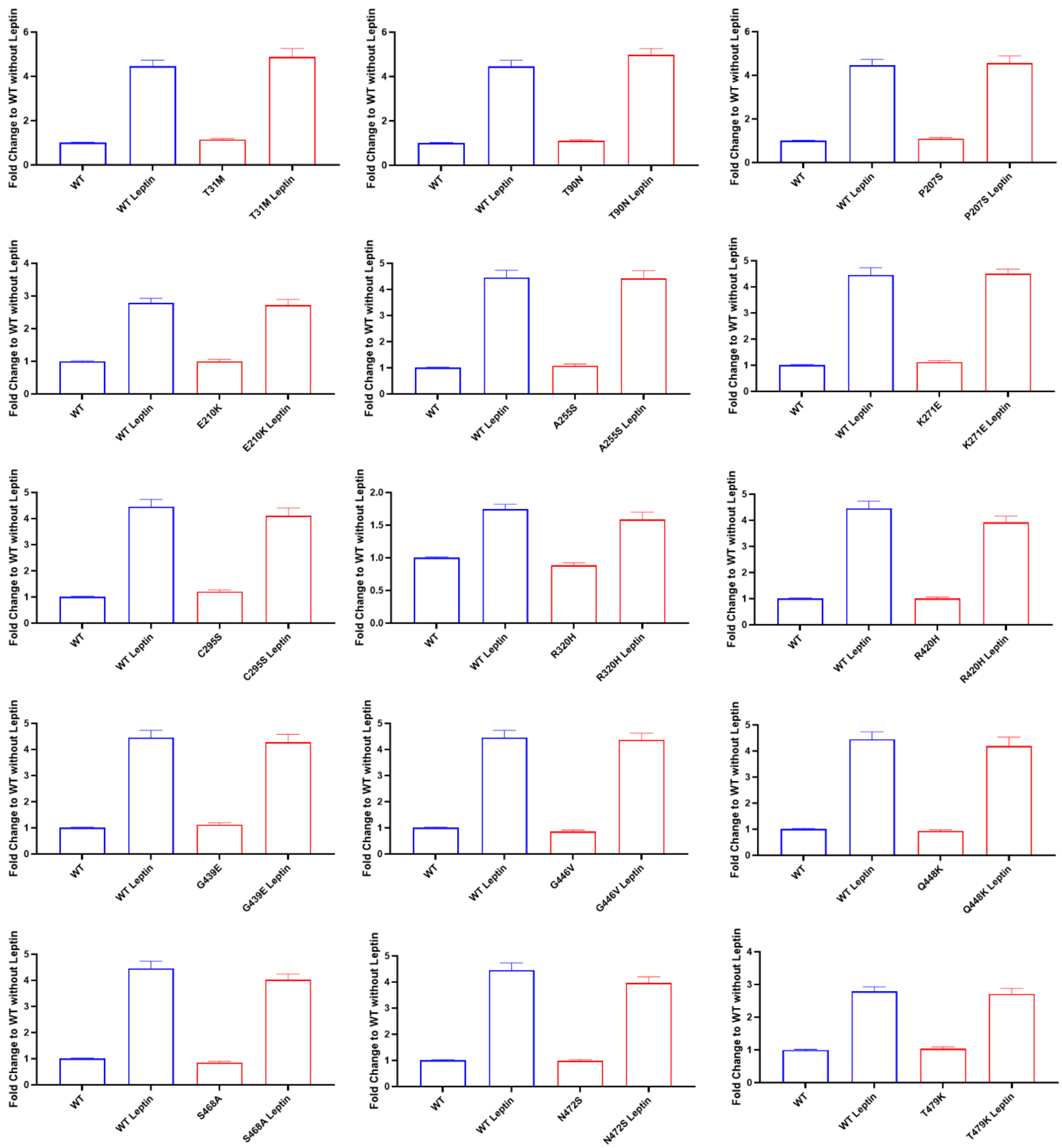

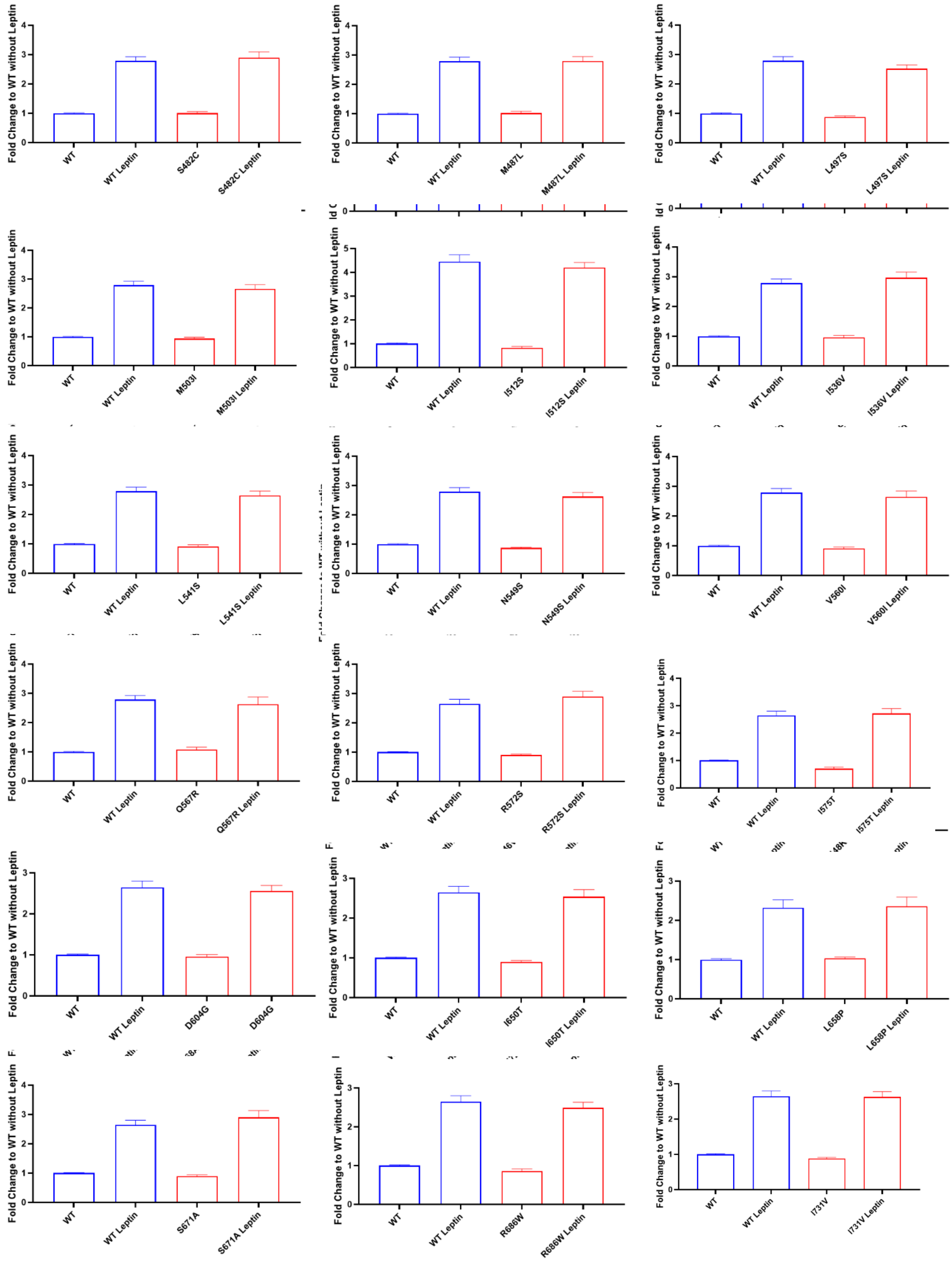
Fold Change to WT without Leptin
WT
WT Leptin
S482C
S482C Leptin
M487L
M487L Leptin
L497S
L497S Leptin
M503I
M503I Leptin
I512S
I512S Leptin
I536V
I536V Leptin
L541S
L541S Leptin
N549S
N549S Leptin
V560I
V560I Leptin
Q567R
Q567R Leptin
R572S
R572S Leptin
I575T
I575T Leptin
D604G
D604G
I650T
I650T Leptin
L658P
L658P Leptin
S671A
S671A Leptin
R686W
R686W Leptin
I731V
I731V Leptin

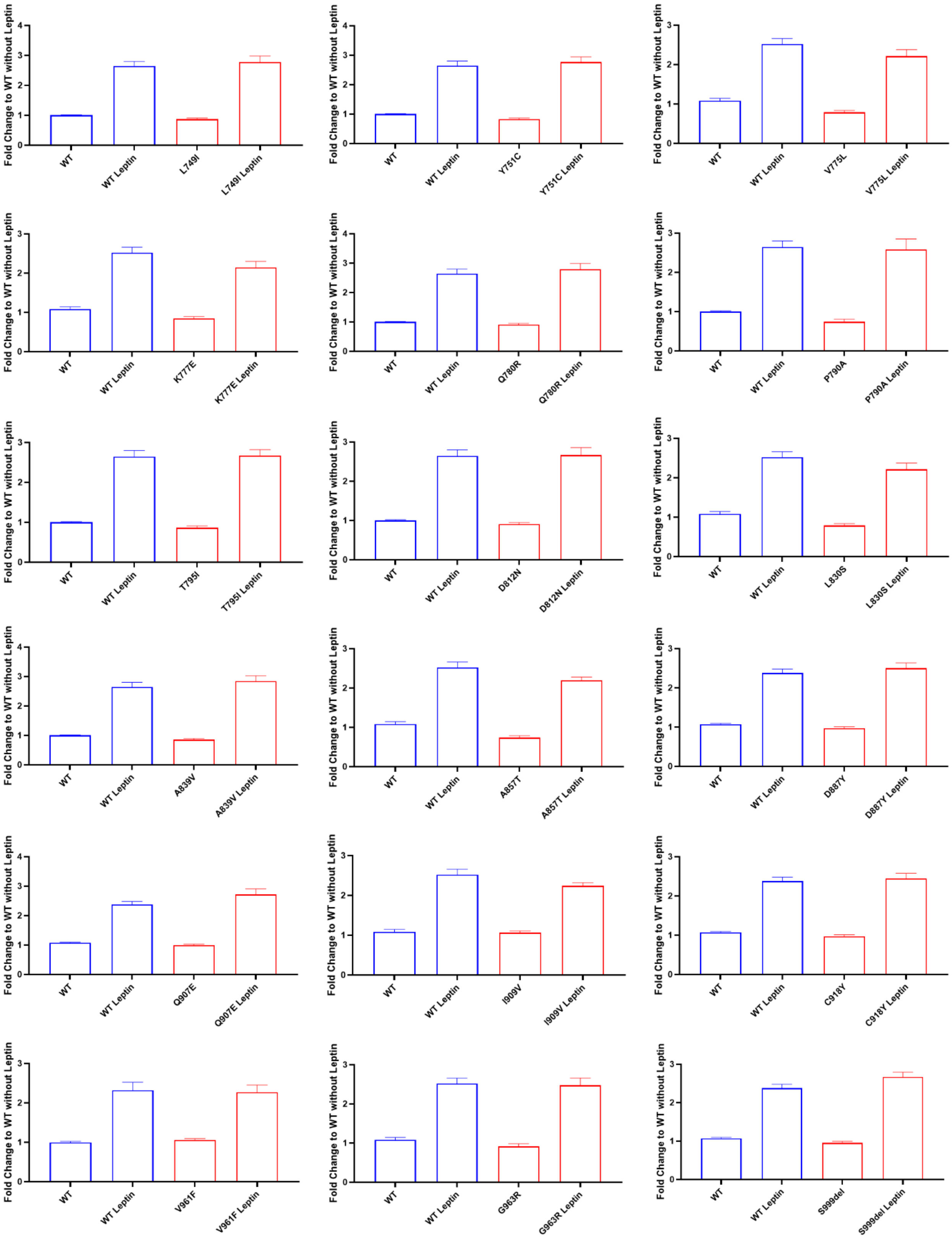

Fold Change to WT without Leptin
WT
WT Leptin
L749I
L749I Leptin
Y751C
Y751C Leptin
V775L
V775L Leptin
K777E
K777E Leptin
Q780R
Q780R Leptin
P790A
P790A Leptin
T795I
T795I Leptin
D812N
D812N Leptin
L830S
L830S Leptin
A839V
A839V Leptin
A857T
A857T Leptin
D887Y
D887Y Leptin
Q907E
Q907E Leptin
I909V
I909V Leptin
C918Y
C918Y Leptin
V961F
V961F Leptin
G963R
G963R Leptin
S999del
S999del Leptin

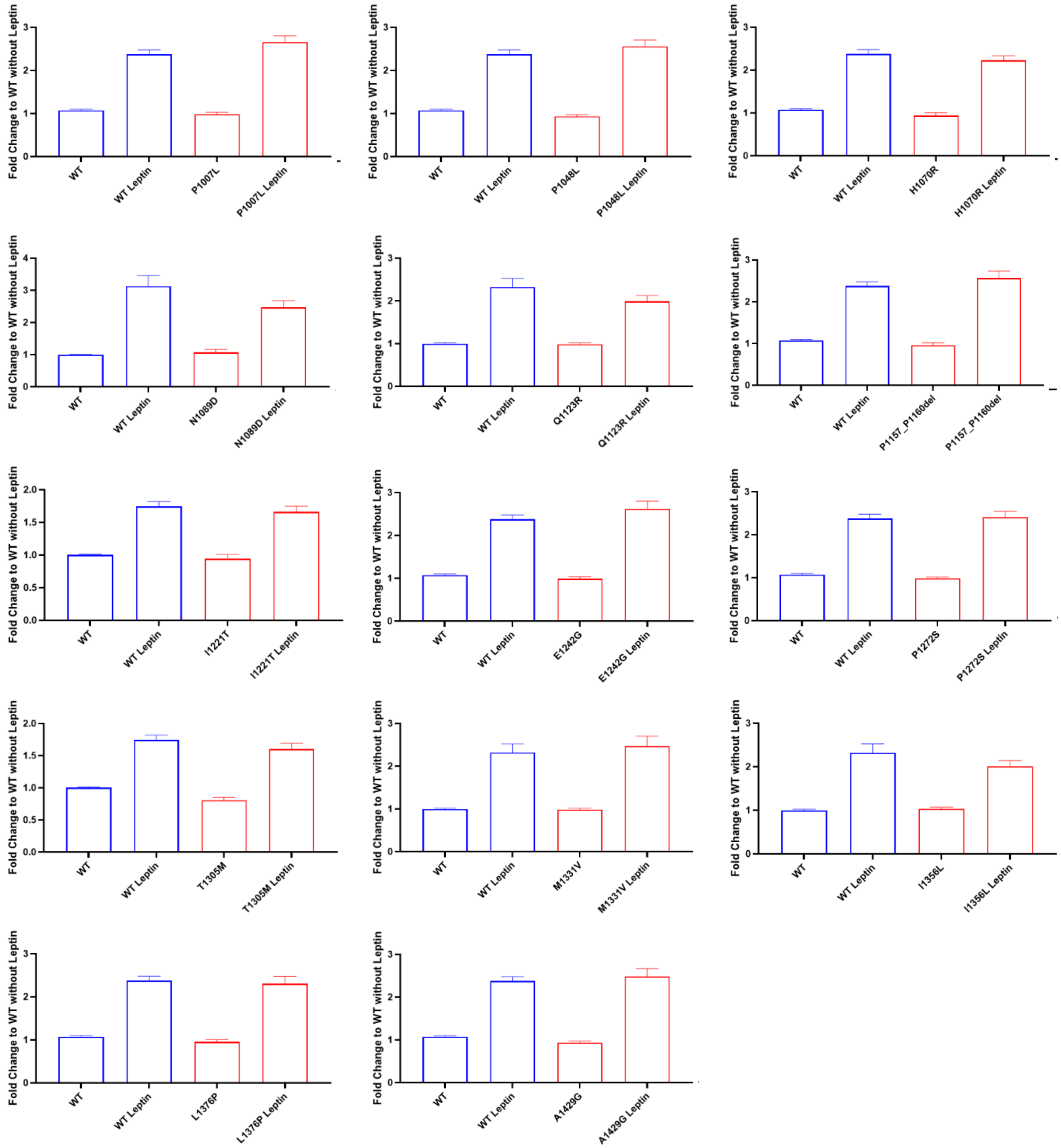

Neuro-2A cells were transiently transfected with a plasmid encoding β-galactosidase for normalization, together with the *NCOA1* expression plasmid (wild-type [WT] or mutant) and the *STAT3*, *LEPR*, and *POMC* promoter reporter constructs. Data are fold changes to luciferase activity of wild-type condition without leptin (mean ± s.e.m.). All experiments were performed in triplicate and each experiment was repeated at least seven times. *P*-values were calculated related to “WT Leptin” by unpaired Mann-Whitney tests.
